# Good practice principles for sharing individual participant data from publicly funded clinical trials

**DOI:** 10.1186/1745-6215-16-S2-O1

**Published:** 2015-11-16

**Authors:** Catrin Tudur Smith, Carolyn Hopkins, Matt Sydes, Kerry Woolfall, Mike Clarke, Gordon Murray, Paula Williamson

**Affiliations:** 1 North West HTMR, University of Liverpool, Liverpool, UK; 2 University of Edinburgh, Edinburgh, UK; 3 All Ireland HTMR, Queen's University, Belfast, UK; 4 London HTMR, MRC Clinical Trials Unit, London, UK

## 

Sharing Individual Participant Data (IPD) from completed clinical trials offers numerous well recognised advantages that can advance clinical research and benefit patients. The clinical trial community, including publicly funded trials, has a duty to facilitate this process.

In our recent survey, publicly funded clinical trials units (CTUs) from the UKCRC registered CTUs network were supportive of the principle of sharing IPD. However, concerns were also raised including complex and varied sponsorship arrangements of the trials they coordinate, inappropriate reuse of clinical trial data, additional resource required for CTUs to prepare and share data, potential loss of ability to publish further research, and the potential risk to trial participant privacy. The CTUs preferred the use of a controlled access approach, with systems in place to review data access requests from researchers.

We have used the results of this survey, input from an expert committee and an open consultation involving the UKCRC registered CTUs to inform the development of a guidance document summarising good practice principles for sharing IPD and associated documentation from publicly-funded clinical trials. The guidance has been endorsed by Cancer Research UK, MRC Methodology Research Programme Advisory Group, Wellcome Trust and the Executive Group of the UK CRC Registered CTUs Network. The National Institute for Health Research (NIHR) has confirmed it is supportive of the application of this guidance.

